# Dose-dependent adverse events of esketamine in treatment-resistant depression: a systematic review and meta-analysis of randomized controlled trials

**DOI:** 10.3389/fphar.2026.1792570

**Published:** 2026-05-28

**Authors:** Yang Qu, Shujin Li, Li Tian, Xiaoxiao Tian, Yongkang Wu

**Affiliations:** 1 West China Hospital Sichuan University Jintang Hospital, Jintang First People’s Hospital, Anesthesia and Surgery Center, Jintang, Sichuan, China; 2 West China Hospital Sichuan University Out-Patient Department, Chengdu, Sichuan, China

**Keywords:** dose-dependent, esketamine, meta-analysis, safety, treatment-resistant depression

## Abstract

**Introduction:**

This study systematically evaluated the safety profile of esketamine for treatment-resistant depression through a meta-analysis, focusing on dose-dependent adverse events and associated risk factors to inform precision dosing.

**Methods:**

PubMed, Embase, the Cochrane Library, the Mainland China Biomedical Literature Database (CBM), the China National Knowledge Infrastructure (CNKI) and Wanfang databases were searched from inception to March 2025. Randomized controlled trials evaluating esketamine for treatment-resistant depression were included. Primary outcomes included the incidence of adverse events, discontinuation due to adverse events, and clinical response or remission. Statistical analysis was conducted using RevMan 5.4.1, with subgroup analyses by dosage, administration route, and geographic region (Mainland China vs. International multi-regional).

**Results:**

Nine randomized controlled trials involving 1,449 patients were included. Dosages ranged from 28 to 84 mg for nasal spray and 0.20–0.40 mg/kg for intravenous injection. Esketamine significantly increased the risk of nine adverse events, including nausea, dissociation, dizziness, vertigo, elevated blood pressure, and somnolence, compared with controls (P < 0.05). Risks were strongly dose-dependent: the high-dose group (≥56 mg or 0.40 mg/kg) showed a greater risk than the low-dose group (≤28 mg or 0.20 mg/kg), with RR for nausea of 3.72 versus 1.69 and RR for dissociation of 10.65 versus 3.27. Patients in International multi-regional studies also had higher risks of nausea, somnolence, and headache than those in Mainland China studies. Although esketamine improved the clinical response rate (RR = 1.94), it increased treatment discontinuation due to adverse events by 2.22-fold (P = 0.025).

**Discussion:**

Esketamine improves symptoms in patients with treatment-resistant depression but significantly increases dose-dependent adverse events. Clinical use should adopt personalized dosing strategies that balance efficacy and tolerability based on individual patient profiles.

**Systematic Review Registration:**

https://www.crd.york.ac.uk/prospero/display_record.php?RecordID=1024830, identifier CRD420251024830.

## 1 Introduction

Treatment-resistant depression (TRD) is a common and highly complex disorder. Treatment-resistant depression (TRD) is typically defined as a failure to respond to at least two antidepressant treatments of adequate dose and duration during the current depressive episode. It affects approximately 30% of patients with major depressive disorder, posing a significant burden on global public health. Patients continue to experience significant symptoms after standard antidepressant treatment fails, imposing a heavy burden on individuals and the healthcare system (Popova et al., 2019; Mcintyre et al., 2021; Martinotti et al., 2022). Traditional antidepressants such as SSRIs or SNRIs have limited efficacy, with a remission rate of less than 30%. New intervention strategies are urgently needed (Popova et al., 2019; Liu et al., 2022).

Esketamine, the S-isomer of ketamine, acts as a high-affinity, non-competitive N-methyl-D-aspartate (NMDA) receptor antagonist,it has a rapid antidepressant effect through its action on NMDA receptors and has been approved by the FDA for the treatment of TRD (Popova et al., 2019; Hope et al., 2023). However, several recent systematic reviews and meta-analyses have evaluated the general safety and efficacy of esketamine (Liu et al., 2022; Nikolin et al., 2023; Reif et al., 2023; Rodolico et al., 2024; Seshadri et al., 2024; Guo et al., 2025), yet it is associated with transient adverse events (AEs) such as dizziness, nausea, dissociative symptoms, and sensory abnormalities, as well as controversy regarding dose-dependent effects (Hock et al., 2022; Yang et al., 2022; Taillefer de Laportalière et al., 2023). This limits the optimization of clinical treatment decisions. Systematic reviews and meta-analyses can effectively reveal dose-dependent safety patterns (Oraee et al., 2024; Ouyang and Li, 2025), Previous meta-analyses have shown that esketamine significantly improves depression scores (such as reductions in MADRS scores) (Liu et al., 2022; Ouyang and Li, 2025), but treatment is accompanied by dizziness, nausea, dissociation, and increased risk of elevated blood pressure (Gastaldon et al., 2021; Ionescu et al., 2021; Zorn et al., 2021; Yang et al., 2022), and dose-specific evidence remains limited (Hock et al., 2022). Differences in safety between administration routes (such as nasal spray vs. intravenous injection) and across regions also require further quantification (Takahashi et al., 2021; Zorn et al., 2021; Zaki et al., 2023).

While previous syntheses have summarized the overall incidence of AEs (Nikolin et al., 2023; Rodolico et al., 2024), they often treated esketamine doses as pooled categories, leaving a gap in understanding precise dose-response thresholds for specific risks. The dose-response relationship regarding adverse events (AEs) remains poorly characterized. Quantifying this relationship is essential for ‘precision dosing’ to balance therapeutic benefits against risks. This study advances beyond previous work by conducting a dose-focused analysis (categorizing doses into specific thresholds: ≥56 mg or 0.40 mg/kg vs. ≤28 mg or 0.20 mg/kg), enabling identification of dose-dependent patterns for symptoms such as nausea and dissociation.

Furthermore, our stratification approach comparing Mainland China and International multi-regional studies provides insight into potential ethnic or regional differences in tolerability, an aspect overlooked in previous reviews. Stratification by geographic region (e.g., China vs. global) is essential, as ethnic variations in drug metabolism—potentially linked to cytochrome P450 enzyme polymorphisms—may influence the incidence and severity of adverse events (AEs). This refined analysis provides clinicians with more tailored evidence-based guidance for dose titration and regional safety monitoring.

## 2 Methods

Trial registration: PROSPERO registration number: CRD 420251024830.

### 2.1 Search strategy

PubMed, Embase, the Cochrane Library, the Mainland China Biomedical Literature Database (CBM), the China National Knowledge Infrastructure (CNKI), and Wanfang Medical Network were searched electronically. Based on these searches, manual searches and reference tracking of included studies were also conducted. The search period covered database inception to March 2025. Mainland China and English search terms included “esketamine,” “treatment-resistant depression,” and “major depressive disorder.“A combination of these terms was used with Boolean operators (AND/OR). The full search strategy for all databases is provided in Supplementary Material S1.

### 2.2 Inclusion and exclusion criteria

#### 2.2.1 Inclusion criteria

1. Study Design: For studies that included both a double-blind randomized phase and an open-label extension phase, only data from the double-blind randomized phase were extracted for quantitative synthesis to avoid selection and performance bias associated with non-randomized designs.
2. Population: Patients with TRD aged 18 years or older without major medical conditions (such as severe cardiovascular diseases).
3. Intervention: Clearly reported doses of esketamine (such as nasal spray 28 mg, 56 mg, or 84 mg, intravenous injection 0.20 mg/kg or 0.40 mg/kg).
4. Control: Placebo, other antidepressants, or different esketamine dose groups.
5. Language: Mainland China and English literature.

#### 2.2.2 Exclusion criteria

1. Non-randomized studies (such as case reports and observational studies).
2. Mixed populations (such as bipolar depression or postpartum depression).
3. Studies with incomplete data or where dose-specific safety data could not be extracted.

### 2.3 Outcome measures

Adverse events (AEs) were evaluated based on their clinical relevance and reporting frequency across the included studies. We pre-specified 11 categories of common AEs for analysis, including dissociation, dizziness, headache, nausea, vomiting, etc. These categories were aligned with the Medical Dictionary for Regulatory Activities (MedDRA) System Organ Class (SOC) to ensure standardized reporting and clinical interpretability. Adverse events reported in the original studies were standardized according to MedDRA terms for consistent reporting (e.g., ‘somnipathy’ was categorized as ‘sleep disorders'.

The secondary outcomes of this study included clinical efficacy measures, specifically: (1) Response rate, defined as a ≥50% reduction from baseline in Montgomery-Åsberg Depression Rating Scale (MADRS) or Patient Health Questionnaire-9 (PHQ-9) scores; and (2) Remission rate, defined as achieving a total score below a pre-specified threshold (e.g., MADRS ≤10).

Serious Adverse Events (SAEs) were defined according to the International multi-regional Council for Harmonisation (ICH) guidelines as any untoward medical occurrence that at any dose results in death, is life-threatening, requires inpatient hospitalization or prolongation of existing hospitalization, or results in persistent or significant disability/incapacity.

Subgroup analyses were pre-specified based on dosage, administration route, and geographic region. The dosage thresholds (e.g., <56 mg, 56 mg, and 84 mg) were selected to align with the standard induction and maintenance doses approved by regulatory agencies and utilized in major clinical trials, allowing for cross-study comparability. The stratification by geographic region (China vs. other countries) was implemented due to several physiological and clinical considerations. First, ethnic-specific genetic polymorphisms in metabolic enzymes, such as CYP2B6, have been shown to influence the pharmacokinetics of esketamine, potentially leading to disparate drug exposure levels between East Asian and Western populations. Second, differences in average body weight between Mainland China and International multi-regional cohorts may result in higher weight-adjusted dosages when fixed-dose protocols are applied, thereby influencing the safety profile. Finally, cultural and clinical differences in the perception and reporting of psychiatric adverse events, such as dissociation, necessitate a localized evaluation to inform precision dosing in the Mainland China clinical context.

### 2.4 Study selection and data extraction

Two researchers independently screened the retrieved literature and removed duplicates by reviewing titles and abstracts. Full texts were then examined to determine eligibility according to the inclusion and exclusion criteria. Disagreements were resolved through discussion, and when necessary, a third reviewer was consulted for arbitration.

Extracted data included the first author and publication year, study design type, diagnosis of the included population, patient medication and dosage, and the number of cases of general adverse reactions (nausea, dizziness, abnormal sensation, dissociative symptoms, sensory dullness, elevated blood pressure, drowsiness, taste disorder, sleep disorder, and physical fatigue).

### 2.5 Quality evaluation

Two evaluators assessed the methodological quality of the included studies based on the Cochrane risk of bias assessment criteria (Higgins et al., 2011).

The Grading of Recommendations Assessment, Development, and Evaluation (GRADE) methodology was used to assess the quality of evidence for each outcome (Guyatt et al., 2011). After the assessment, a GRADE evidence profile table was created to rate the quality of all outcomes.

### 2.6 Statistical analysis

A meta-analysis was conducted using RevMan 5.4.1. All effect sizes were expressed as RR with 95% CI. Heterogeneity was assessed using the I^2^ statistic and tau^2^. For outcomes with I^2^ > 50%, a random-effects model was applied. Additionally, 95% prediction intervals (PIs) were calculated to evaluate the distribution of true effects across settings. Sensitivity analysis was conducted using the leave-one-out method to evaluate the stability of pooled results. A difference was considered statistically significant when P < 0.05. Subgroup analyses were also conducted according to esketamine dose (low dose: ≤28 mg or 0.20 mg/kg; high dose: ≥56 mg or 0.40 mg/kg), administration route (nasal spray or intravenous injection), and geographic region (Mainland China vs. International multi-regional studies).

## 3 Results

### 3.1 Search results and study characteristics

#### 3.1.1 Study retrieval and selection

**FIGURE 1:**
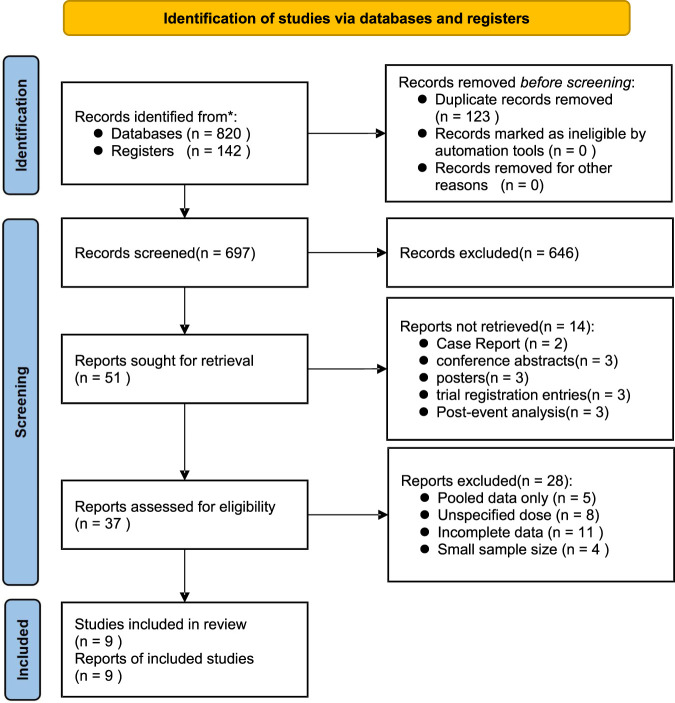
PRISMA flow diagram. PRISMA = Preferred Reporting Items for Systematic Reviews and Meta-Analyses.

This systematic review and meta-analysis was conducted in accordance with the Preferred Reporting Items for Systematic Reviews and Meta-Analyses (PRISMA) 2020 statement (Page et al., 2021). The search process and study selection are summarized in Figure 1. After removing duplicates (n = 123) and excluding articles unrelated to the research question during title and abstract screening (n = 646), 51 articles remained for full-text screening. Of these, 28 studies were excluded because they did not meet the predefined inclusion criteria, and 14 studies could not be retrieved. Finally, nine articles involving 1,449 patients were included in the analysis.

#### 3.1.2 Study characteristics

The characteristics of the included studies are summarized in Table 1. Nine studies were included (Singh et al., 2016; Daly et al., 2018; Daly et al., 2019; Fedgchin et al., 2019; Ochs-Ross et al., 2020; Takahashi et al., 2021; Han, 2022; Zeng, 2022; Chen et al., 2023).

**TABLE 1:** The characteristics of the included studies.

study	Sample	Intervention information experimental/control group		Follow-up period	Evaluation criteria
Daly et al. (2018)	89	ES(NS)	P	8 w	②④⑤⑥
Daly et al. (2019)	297	ES(NS)+A	P + A	2 w	②④⑤⑥
Han (2022)	58	ES(IV)	P	4 h	①⑥
Singh et al. (2016)	30	ES(IV)	P	4 w	②④⑤⑥
Fedgchin et al. (2019)	344	ES(NS)+A	P + A	24 w	②④⑤⑥
Takahashi et al. (2021)	202	ES(NS)+A	P + A	24 w	②④⑤⑥
Ochs-Ross et al. (2020)	137	ES(NS)+A	P + A	2w	②④⑤⑥
Chen et al. (2023)	252	ES(NS)+A	P + A	8 w	②④⑥
Zeng et al. (2022)	40	ES(IV)	PROP	4 h	①②③④⑤⑥

ES: esketamine; A: antidepressants; P: placebo; NS: nasal spray; IV: intravenous injection; PROP: propofol, ① Hamilton Depression Scale – 17 (HAMD-17); ② Montgomery-Åsberg Depression Rating Scale ((MADRS) ③ Hamilton Anxiety Scale (HAMA); ④ Treatment discontinuation rate; ⑤ Remission rate (MADRS, score ≤12 or HAMD-17, score ≤7); ⑥ Evaluation of adverse reactions.

#### 3.1.3 Risk of bias assessment

**FIGURE 2:**
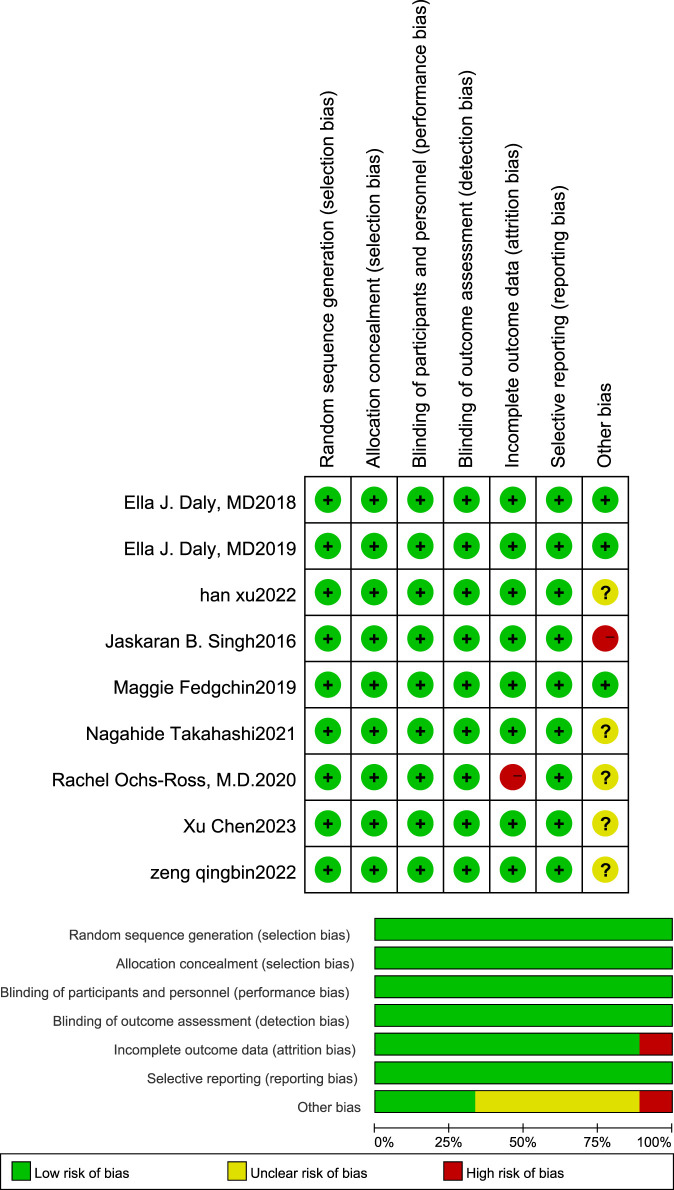
Risk of bias assessment results for individual studies.

The quality evaluation of the included studies is presented in Table 2; Figure 2. As shown in Table 2, all included studies were of high quality. Although two domains showed high risk of bias (incomplete outcome data, and other biases) these factors did not significantly affect the results of this study.

**TABLE 2:** Each risks of bias item for each study.

study	Random sequence generation (selection bias)	Allocation concealment (selection bias)	Blinding of participants and personnel (performance bias)	Blinding of outcome assessment (detection bias)	Incomplete outcome data (attrition bias)	Selective reporting (reporting bias)	Other bias
Daly et al. (2018)	Low	Low	Low	Low	Low	Low	Low
Daly et al. (2019)	Low	Low	Low	Low	Low	Low	Low
Han (2022)	Low	Low	Low	Low	Low	Low	Unclear
Singh et al. (2016)	Low	Low	Low	Low	Low	Low	High
Fedgchin et al. (2019)	Low	Low	Low	Low	Low	Low	Low
Takahashi et al. (2021)	Low	Low	Low	Low	Low	Low	Unclear
Ochs-Ross et al. (2020)	Low	Low	Low	Low	High	Low	Unclear
Chen et al. (2023)	Low	Low	Low	Low	Low	Low	Unclear
Zeng et al. (2022)	Low	Low	Low	Low	Low	Low	Unclear

### 3.2 Overall safety and efficacy analysis (experimental vs. control)

#### 3.2.1 Analysis of adverse reactions of esketamine

The adverse events selected for this study cover the common safety outcomes associated with esketamine, including psychiatric disorders, nervous system disorders, and other sensory disorders. Psychiatric disorders mainly manifest as dissociative symptoms, drowsiness, and sleep disorders. Nervous system disorders mainly manifest as headache, dizziness, and vertigo. Other sensory disorders mainly manifest as taste disorders, nausea, abnormal sensations, physical fatigue, and elevated blood pressure.

**FIGURE 3:**
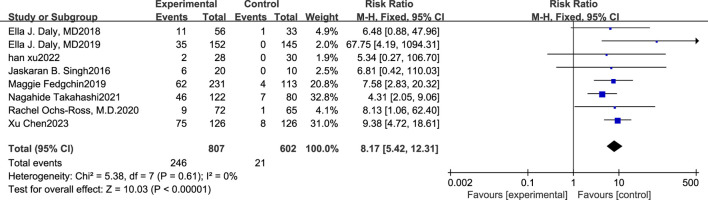
Forest plot of dissociative symptoms.

**FIGURE 4:**
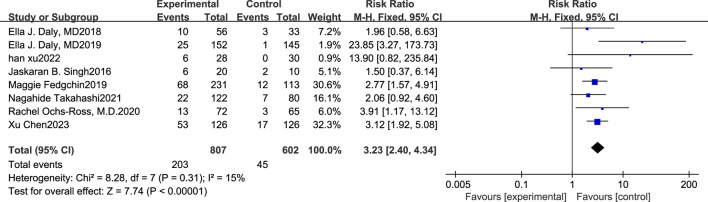
Forest plot of nausea.

**FIGURE 5:**
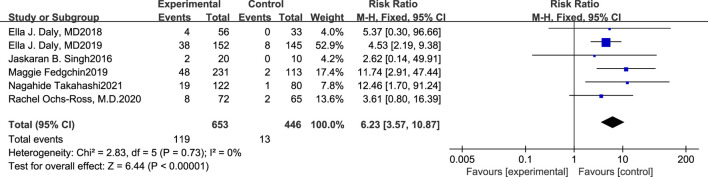
Forest plot of Vertigo.

**FIGURE 6:**
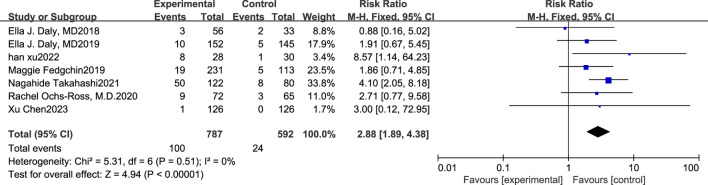
Forest plot of elevated blood pressure.

Our meta-analysis evaluated 11 categories of adverse events (AEs). Esketamine treatment was associated with a significantly higher risk in nine of 11 AEs compared with the control group (Table 3; Figures 3–6). Due to the large number of outcomes and space limitations, only a portion of the adverse reaction forest plots are presented. The remaining figures are provided in the Supplementary Material.

**TABLE 3:** Adverse reactions of esketamine.

adverse reaction	Number of adverse reactions/total number experimental/control group		Zheterogeneity	Z	P	RR	95%CI	τ Martinotti et al. (2022)	95% PI
Nausea	203/807	45/602	χ2 = 8.28 P = 0.31, I^2^ = 15%	7.74	<0.00001	3.23	2.4, 4.34	0.08	1.65–7.51
Paresthesia	80/653	8/446	χ2 = 3.28, P = 0.66, I^2^ = 0%	5.16	<0.00001	5.51	2.88, 10.52	0.12	0.78–8.82
Dissociative symptoms	24/807	21/602	χ2 = 5.38, P = 0.61, I^2^ = 0%	10.03	<0.00001	8.17	5.42, 12.31	0.1	2.54–18.42
Fatigue	155/723	55/549	χ2 = 9.64, P = 0.09, I^2^ = 48%	5.22	<0.00001	2.09	1.59, 2.76	0	0.85–2.79
Somnolence	117/525	37/358	χ2 = 12.47, P = 0.006, I^2^ = 76%	1.78	0.07	2	0.93, 4.28	0.58	0.42–30.15
Sleep disorders	34/505	24/357	χ2 = 2.5, P = 0.64, I^2^ = 0%	0.16	0.87	0.96	0.60, 1.54	0	0.65–2.28
Headache	150/827	66/622	χ2 = 6.48, P = 0.59, I^2^ = 0%	3.69	0.0002	1.65	1.26, 2.15	0.01	0.98–1.86
Dizzy	286/827	62/622	χ2 = 8.31, P = 0.4, I^2^ = 4%	10.32	<0.00001	3.69	2.88, 4.74	0.18	1.42–11.95
Dysgeusia	99/827	38/562	χ2 = 14.64, P = 0.01, I^2^ = 66%	1.13	0.26	1.54	0.73, 3.27	0.32	0.48–16.85
Vertigo	119/653	13/446	χ2 = 2.83, P = 0.73, I^2^ = 0%	6.44	<0.00001	6.23	3.57, 10.87	0.15	1.25–17.32
Elevated blood pressure	100/787	24/592	χ2 = 5.31, P = 0.51, I^2^ = 0%	4.94	<0.00001	2.88	1.89, 4.38	0.09	0.82–6.91

Dissociation exhibited the highest increase in risk (RR = 8.17, 95% CI: 5.42–12.31), with a 95% PI of 2.54–18.42, indicating a consistently elevated risk across clinical settings. Other significant neurological and gastrointestinal AEs included dizziness (RR = 3.69), vertigo (RR = 6.23), somnolence (RR = 2.00), and nausea (RR = 3.23).

Substantial heterogeneity was observed for somnolence (I^2^ = 76%) and dysgeusia (I^2^ = 66%). For these outcomes, the 95% PIs crossed the line of no effect (1.0), suggesting that the incidence may vary depending on administration route or dosing flexibility. In contrast, headache showed the lowest heterogeneity (I^2^ = 0), indicating a stable safety finding. No significant differences were observed between groups for fatigue (I^2^ = 48%) and sleep disorders (I^2^ = 0).

#### 3.2.2 Analysis of the discontinuation rate of esketamine treatment

**FIGURE 7:**
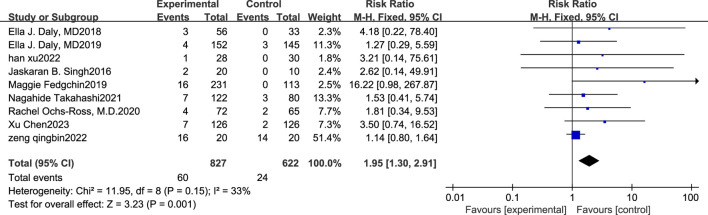
Forest plot of discontinuation rate for esketamine treatment.

The risk of treatment discontinuation was significantly higher in the esketamine group was than in the control group (overall P = 0.001). For the treatment discontinuation analysis, we distinguished between all-cause discontinuation and discontinuation due to adverse events (DAE) (Table 4). The risk of DAE was significantly higher in patients receiving esketamine (RR = 2.22, 95% CI: 1.11–4.45, P = 0.025). This finding suggests that treatment discontinuation in the experimental group was primarily driven by tolerability issues rather than inefficacy or other factors. The discontinuation rate of esketamine treatment is shown in Figure 7.

**TABLE 4:** Discontinuation rates of esketamine treatment.

Outcome	No. Of RCTs	RR (95% CI)	P-value	I Martinotti et al. (2022)
All-cause Discontinuation	9	1.15 (0.85, 1.56)	0.36	0%
Discontinuation due to AEs	9	2.22 (1.11, 4.45)	0.025	0%

#### 3.2.3 Analysis of remission rate with esketamine

**FIGURE 8:**
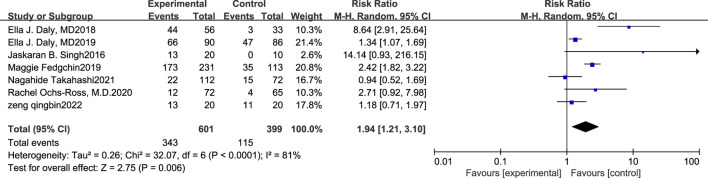
Forest plot of response rate for esketamine treatment.

Regarding clinical efficacy, esketamine significantly improved response rates in patients with treatment-resistant depression compared with the placebo group (RR = 1.94, 95% CI: 1.21–3.10). Despite the overall positive effect, high heterogeneity was observed (I^2^ = 81%), and the 95% PI (0.51–4.30) crossed the line of no effect, indicating that the magnitude of the treatment effect may vary considerably across clinical settings or future studies. The response rate of esketamine treatment is shown in Figure 8.

#### 3.2.4 Analysis of serious adverse reactions of esketamine

**FIGURE 9:**
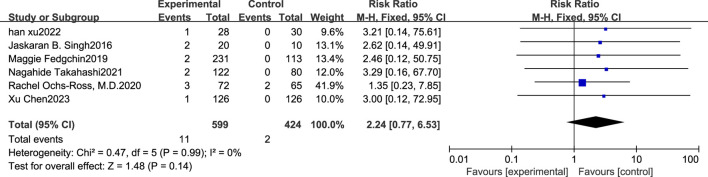
Forest plot of severe adverse reactions of esketamine.

There was no significant difference between the two groups (P = 0.14). The serious adverse events (SAEs) of esketamine are shown in Figure 9.

### 3.3 Pre-specified subgroup analyses

**FIGURE 10:**
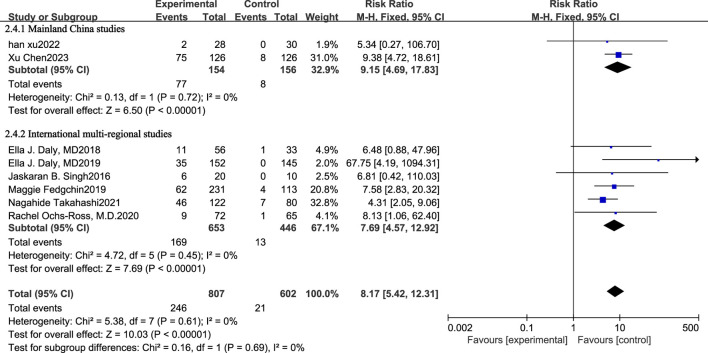
Forest plot of subgroup analysis of dissociative symptoms in Mainland China and International multi-regional studies.

**FIGURE 11:**
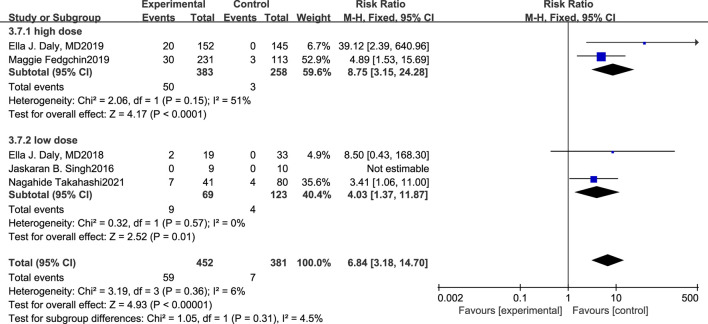
Forest plot of dose subgroup analysis for esketamine paresthesia.

**FIGURE 12:**
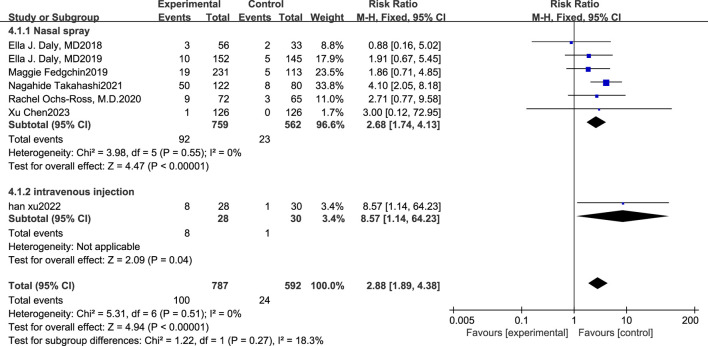
Forest plot of elevated blood pressure with esketamine by route of administration.

To further explore the sources of heterogeneity and identify potential risk factors, we conducted pre-specified subgroup analyses based on dose tier, geographic region, and administration route (Tables 5–8; Figures 10–12).

**TABLE 5:** Subgroup analysis by dose.

Adverse reaction	low dose (≤28 mg or 0.20 mg/kg) RR (95%CI) p I Martinotti et al. (2022) (%)	high dose (≥56 mg or 0.40 mg/kg) RR (95%CI) p I Martinotti et al. (2022) (%)
Nausea	1.69 (0.81, 3.55) 0.16 48	3.72 (2.6, 5.32) <0.00001 0
Paresthesia	4.03 (1.37, 11.87) 0.01 51	8.75 (3.15, 24.28) 0.01 0
Dissociation symptoms	3.27 (1.54, 6.93) 0.002 0	10.65 (6.2, 18.29) <0.00001 0
Fatigue	1.39 (0.68, 2.86) 0.37 68	2.3 (1.24, 4.26) 0.008/
Somnolence	1.39 (0.68, 2.86) 0.37 84	2.3 (0.67, 7.94) 0.19/
Sleep disorders	0.57 (0.02, 13.26) 0.72 19	0.92 (0.55, 1.53) 0.75/
Headache	2.88 (1.31, 6.36) 0.009 0	1.41 (1.05, 1.89) 0.02 0
Dizziness	4.61 (1.95, 10.89) 0.0005 17	3.39 (2.59, 4.43) <0.00001 0
Dysgeusia	2.03 (0.08, 54.57) 0.67 81	1.65 (0.5, 5.43) 0.41 76
Vertigo	8.05 (1.4, 6.35) 0.02 36	6.31 (3.26, 12.22) <0.00001 0
Elevated blood pressure	2.27 (1.07, 4.79) 0.03 0	2.41 (1.28, 4.55) 0.007 48

#### 3.3.1 Dose-dependent patterns of adverse events

Our analysis revealed distinct dose-response relationships for several key adverse events (Table 5).

Dissociation and Nausea: These symptoms showed the most pronounced dose-dependency. The risk of dissociation was significantly higher in the high-dose group (RR = 10.65, 95% CI: 6.20–18.29, P < 0.00001) than in the low-dose group (RR = 3.27, 95% CI: 1.54–6.93, P = 0.002). Similarly, nausea reached statistical significance only in high-dose treatments (RR = 3.72, 95% CI: 2.60–5.32, P < 0.00001), whereas low-dose treatments showed no significant difference compared to controls (RR = 1.69, 95% CI: 0.81–3.55, P = 0.16).

Headache: Interestingly, while both groups exhibited an increased risk, the effect size was larger in the low-dose group (RR = 2.88, 95% CI: 1.31–6.36, P = 0.009) compared to the high-dose group (RR = 1.41, 95% CI: 1.05–1.89, P = 0.02).

#### 3.3.2 Geographic and ethnic variations

Stratification by geographic region (Mainland China vs. International multi-regional studies) highlighted potential ethnic differences in tolerability (Table 6):

**TABLE 6:** Subgroup analysis of Mainland China and international multi-regional studies.

Adverse reaction	Mainland China RR (95%CI) P I Martinotti et al. (2022) (%)	International multi-regional RR (95%CI) P I Martinotti et al. (2022) (%)
Nausea	2.24 (0.09, 53.13) 0.62 80	2.74 (1.66, 4.52) <0.0001 28
Dissociation symptoms	9.15 (4.69, 17.83) <0.00001 0	7.69 (4.57, 12.92) <0.00001 0
Fatigue	1.75 (1.11, 2.76) 0.02 0	2.26 (1.6, 3.18) <0.00001 68
Somnolence	0.86 (0.35, 2.1) 0.74/	2.59 (1.07, 6.25) 0.03 78
Sleep disorders	1.11 (0.55, 2.24) 0.78 41	0.89 (0.48, 1.65) 0.72 0
Headache	1.35 (0.85, 2.16) 0.2 0	1.78 (1.29, 2.46) 0.0005 0
Dizziness	3.43 (2.5, 4.7) <0.00001 53	3.96 (2.71, 5.79) <0.00001 0
Dysgeusia	0.33 (0.01, 8.11) 0.5/	1.67 (0.76, 3.64) 0.2 71
Elevated blood pressure	6.67 (1.25, 35.51) 0.03 0	2.67 (1.73, 4.13) <0.00001 0

Neurological Symptoms: The risk of dissociation was consistently high across both Mainland China (RR = 9.15, 95% CI: 4.69–17.83, P < 0.00001) and International multi-regional trials (RR = 7.69, 95% CI: 4.57–12.92, P < 0.00001). Dizziness followed a similar pattern (Mainland China: RR = 3.96; International multi-regional: RR = 3.43).

Cardiovascular and Systemic Effects: Mainland China populations appeared more sensitive to blood pressure elevation (RR = 6.67, 95% CI: 1.25–35.51, P = 0.03) compared to International multi-regional cohorts (RR = 2.67, 95% CI: 1.73–4.13, P < 0.00001). Conversely, International multi-regional studies reported a higher risk of fatigue (RR = 2.26 vs. 1.75 in Mainland China studies) and nausea (RR = 2.74, P < 0.0001 vs. RR = 2.24, P = 0.62 in Mainland China studies).

Subgroup analysis based on geographic region demonstrated that esketamine was effective in Western/global (RR = 1.56, P = 0.008) whereas the effect in Asian populations was not statistically significant (RR = 1.18, P = 0.251). However, the interaction test (P = 0.208) indicated no significant efficacy difference between these groups, suggesting that ethnicity does not sufficiently explain the overall heterogeneity (I^2^ = 81%). The subgroup analysis of remission by geographic region (ethnicity) is shown in Table 7.

**TABLE 7:** Subgroup analysis of remission by geographic region (ethnicity).

Subgroup	No. Of Studies	Esketamine (n/N)	Control (n/N)	RR (95% CI)	P-value	I^2^ (%)
Western/Global	5	166/493	69/334	1.56 (1.12, 2.16)	0.008	36.40%
Asian (China/Japan)	4	63/199	48/158	1.18 (0.89, 1.56)	0.251	0.00%
Overall	9	229/692	117/492	1.94 (1.21, 3.10)	0.001	3.60%
Test for Subgroup Difference	​	​	​	P = 0.208	​	​

#### 3.3.3 Impact of administration route

When focusing on the intranasal route (the most common delivery method), esketamine remained significantly associated with nearly all recorded AEs (P < 0.05), with the notable exception of sleep disorders (RR = 0.81, 95% CI: 0.45–1.48, P = 0.49).

These findings confirm that the intranasal route, while effective, consistently triggers the core safety profile of esketamine across diverse patient populations (Table 8).

**TABLE 8:** Subgroup analysis by route of administration.

Adverse reaction	Nasal spray RR (95%CI) p I Martinotti et al. (2022) (%)	intravenous injection RR (95%CI) p I Martinotti et al. (2022) (%)
Nausea	3.22 (2.37, 4.37) <0.00001 18	3.4 (1.04, 11.1) 0.04 55
Paresthesia	5.68 (2.92, 11.04) <0.00001 0	2.62 (0.14, 49.91) 0.52/
Dissociation symptoms	7.6 (2.13, 27.17) 0.002 83	6.09 (0.79, 46.72) 0.08 0
Fatigue	2.17 (1.59, 2.95) <0.00001 57	1.67 (0.96, 2.88) 0.07/
Somnolence	2.59 (1.07, 6.25) 0.03 78	0.86 (0.35, 2.10) 0.74/
Sleep disorders	0.81 (0.45, 1.48) 0.49 0	1.43 (0.68, 3.00) 0.35/
Headache	1.62 (1.2, 2.18) 0.001 13	1.81 (1.01, 3.24) 0.04 0
Dizziness	3.94 (3.01, 5.16) <0.00001 0	2.19 (1.16, 4.13) 0.02 0
Vertigo	6.39 (3.62, 11.28) <0.00001 0	2.62 (0.14, 49.91) 0.52/
Elevated blood pressure	2.68 (1.74, 4.13) <0.00001 0	8.57 (1.14, 64.23) 0.04/

### 3.4 Sensitivity analysis and exploration of heterogeneity

To investigate the sources of heterogeneity, a leave-one-out sensitivity analysis was conducted for all primary outcomes (Table 9):

**TABLE 9:** Leave-one-out sensitivity analysis.

Study Omitted	Somnolence I Martinotti et al. (2022)	Remission I Martinotti et al. (2022)	Dysgeusia I Martinotti et al. (2022)	Source identification
None	76%	81%	66%	——
Daly et al. (2018)	75%	80%	64%	Have a relatively minor impact
Fedgchin et al. (2019)	74%	79%	61%	Have a relatively minor impact
Singh et al. (2016)	42% ↓	81%	65%	The only IV administration study showed a high peak blood concentration
Ochs-Ross et al. (2020)	76%	55% ↓	66%	The only study on the elderly population showed a relatively low response rate to the treatment
Daly et al. (2019)	68%	76%	32% ↓	The flexible dosage design results in uneven drug exposure
Takahashi et al. (2021)	75%	78%	63%	Data from the Japanese population show that it has a limited contribution to overall heterogeneity

For somnolence: The exclusion of Singh et al. (2016) markedly reduced I^2^ from 76% to 42%, identifying this study as the primary source of variance. This is likely due to its intravenous (IV) administration route compared with the intranasal delivery used in other trials.

For dysgeusia: Excluding Daly et al. (2019) led to a decrease in I^2^ from 66% to 32%. The heterogeneity in this outcome was attributed to the flexible-dose design of the Daly trial, which contrasted with the fixed-dose protocols of the remaining RCTs.

For remission: Excluding Ochs-Ross et al. (2020), which focused exclusively on an elderly population, reduced I^2^ from 81% to 55%. This suggests that age-related differences in pharmacological response contributed to efficacy variance.

## 4 Discussion

### 4.1 Dose-dependent patterns and safety-tolerability trade-off

A key finding of this meta-analysis is the dose-response relationship observed across common adverse events (AEs). We identified three distinct dose-related patterns: (1) a significant dose-dependent increase in symptoms such as dissociation, paresthesia, and nausea; (2) a relatively stable or slightly declining incidence for headache and dizziness at higher doses; and (3) dose-independent effects, such as elevated blood pressure, which require consistent monitoring regardless of dose level. These results align with previous studies showing that dose escalation is associated with increased AE incidence (Dean et al., 2021; Seshadri et al., 2024).

Our analysis of discontinuation due to adverse events (DAE) shows that esketamine entails a 2.22-fold higher risk (RR = 2.22, 95% CI: 1.11–4.45) of treatment cessation compared with placebo. This suggests that the tolerability threshold for esketamine may be narrow in some patients, particularly when escalating from 56 mg to 84 mg (Fedgchin et al., 2019; Popova et al., 2019). From a clinical perspective, this necessitates a shift from a one-size-fits-all approach to individualized titration based on patient tolerance (Lucchese et al., 2021).

### 4.2 Reframing dissociation: toxic reaction vs. pharmacological marker

The nature of dissociative symptoms, the most documented AE in our study, must be understood within a pharmacological context. Dissociation is regarded as a typical acute effect of esketamine rather than a toxic reaction. These symptoms manifest as temporary disturbances in self-perception, usually emerging shortly after administration and subsiding within 1.5 h, demonstrating temporal predictability and self-limitation (Zhou et al., 2024; Kosik-Gonzalez et al., 2026).

Unlike traditional drug toxicity, these symptoms are reversible and do not appear to cause persistent neurocognitive impairment (Arjmand et al., 2025; Kosik-Gonzalez et al., 2026). While dissociation reflects the regulation of the glutamatergic system and the initiation of pharmacological activity, current evidence indicates that its severity does not necessarily correlate with antidepressant response (Medeiros et al., 2024). Thus, dissociation should be understood as a transient pharmacological marker of NMDA receptor antagonism (Bottemanne et al., 2023; Zhou et al., 2024; Arjmand et al., 2025).

### 4.3 Administration routes and heterogeneity in somnolence

The route of administration influences the distribution of AEs. Our results identified the administration route as a driver of heterogeneity, particularly for somnolence (I^2^ = 76%). Sensitivity analysis showed that excluding the study by Singh et al. (2016), the only trial utilizing intravenous (IV) administration, reduced this variance. From a pharmacokinetic (PK) perspective, IV injection bypasses the nasal mucosal barrier, leading to a rapid increase in plasma concentration, which may trigger more acute sedation compared with the gradual absorption of nasal sprays (Piggott et al., 1993).

While nasal sprays are associated with a range of neuropsychiatric treatment-emergent adverse events (TEAEs) due to their bioavailability (Cook and Halaris, 2022; Rosenman, 2022; Scarcella et al., 2024; Seshadri et al., 2024; Chevalier et al., 2025), they may offer better cardiovascular safety in long-term management. However, because the IV evidence is derived from a single study with a small sample size, these route-specific findings should be interpreted with caution as exploratory rather than definitive.

### 4.4 Efficacy across populations: the impact of ethnicity and age

The rationale for the ‘Mainland China vs. International’ stratification is supported by several factors. First, pharmacokinetic (PK) differences are significant; population PK models indicate that Asian subjects have approximately 36% higher esketamine exposure (AUC and C_max_) compared to non-Asian subjects, which is partly attributed to genetic polymorphisms in metabolism enzymes like *CYP2B6* (Perez-Ruixo et al., 2021). Second, body weight serves as a critical covariate. The median body weight in Chinese cohorts is typically lower (60–65 kg) than in Western-dominated international trials (approx. 80 kg), leading to a higher weight-adjusted dose under fixed-dose regimens (NCD-RisC, 2017). Finally, cultural nuances influence the reporting of psychiatric AEs. For instance, Chinese patients may perceive and report dissociative symptoms differently due to cultural shaping of distress expression (Ryder et al., 2008). It is important to note that while this stratification is primarily geographical, it serves as a proxy for these underlying biological and social differences, rather than a strict ‘East Asian vs. Western’ racial dichotomy.

The high heterogeneity observed in remission rates (I^2^ = 81%) was initially suspected to stem from ethnic differences. However, our subgroup analysis indicates that both the efficacy and tolerability of esketamine are largely independent of geographic region (P = 0.208 for remission). This supports the extrapolation of global pivotal trial data to East Asian (Mainland China) populations without the immediate need for major ethnicity-based dose adjustments. This is consistent with PK studies showing that while *CYP2B6* genetic polymorphisms vary by ethnicity, the overall benefit-risk ratio remains stable (Perez-Ruixo et al., 2021; Arjmand et al., 2025).

Instead, age appears to be a critical factor for heterogeneity. Clinical remission variance was mitigated after excluding the study on older adults (Ochs-Ross et al., 2020). This difference likely arises from age-related physiological changes, such as decreased NMDA receptor density and sensitivity, alongside altered drug clearance rates in older adults (Piggott et al., 1993). Additionally, the heterogeneity in dysgeusia (I^2^ = 66%) may be attributed to flexible-dose designs (e.g., Daly et al., 2019), where dynamic adjustments between 56 mg and 84 mg led to inconsistent local spray volumes (Citrome, 2019).

Furthermore, at notable factor contributing to the heterogeneity of our findings is the varied composition of the control groups across the included trials. In our analysis, three studies utilized an inert placebo, while five trials involved patients receiving background antidepressants (ADs) and one utilized other medications as the comparator. This heterogeneity is particularly relevant when evaluating subjective adverse events such as nausea, headache, or somnolence, which are common side effects of traditional antidepressants.

In trials where the control group received an active AD, the baseline incidence of AEs in the control arm was likely higher than in pure placebo-controlled trials. Consequently, the relative risk (RR) for esketamine in these “add-on” studies might be lower (or “diluted”) compared to placebo-controlled designs, as the control group also experienced pharmacological side effects. However, this diversity also reflects the clinical reality of Treatment-Resistant Depression (TRD) management, where esketamine is frequently administered as an adjunctive therapy to existing antidepressant regimens rather than as a monotherapy. While this active-control design may lead to a more conservative estimate of esketamine’s risk profile, it provides higher ecological validity for clinical decision-making. Future meta-analyses with a larger number of trials could benefit from performing a formal meta-regression or separate subgroup analyses based on the type of control (Placebo vs. Active AD) to further isolate the specific toxicological impact of esketamine.

### 4.5 Clinical implications and risk mitigation

The risk of common adverse reactions, such as dissociation and elevated blood pressure, is well documented. Dissociative symptoms were more frequent in the experimental group, especially with nasal spray, which is consistent with a retrospective study of 70 TRD patients (Del sant et al., 2022). Similarly, while tolerance is generally good, open-label data suggest that psychiatric symptoms may occur in some patients (Smith-Apeldoorn et al., 2022).

In summary, while esketamine provides therapeutic benefits (RR = 1.35 for remission), its clinical use requires a careful balance against tolerability risks (RR = 2.22 for DAE). Clinicians should prioritize the 56 mg starting dose to establish tolerability before escalating to 84 mg, particularly in patients who show early signs of severe dissociation or in older populations who may require precise titration. Strict safety protocols, including real-time monitoring of blood pressure and psychiatric symptoms, remain essential to minimize the risk of treatment discontinuation and potential long-term risks such as addiction or suicidal ideation (Horowitz and Moncrieff, 2021; Jawad et al., 2022; Jiang et al., 2025).

## 5 Limitations

Despite the comprehensive nature of this meta-analysis, some limitations should be acknowledged.

First, while we observed a potential impact of administration route on the safety profile, the number of studies utilizing intravenous (IV) delivery was limited, with only one study (Singh et al., 2016) included in this category. This resulted in wide confidence intervals and reduced the statistical power for route-specific comparisons, rendering these findings exploratory.

Second, although we conducted a subgroup analysis between East Asian (Mainland China) and global/Western populations, other ethnic groups, such as African and Hispanic populations, remained underrepresented. Therefore, the findings regarding ethnic consistency in esketamine safety may not be fully generalizable to all racial groups.

Third, most included randomized controlled trials (RCTs) focused on acute-phase treatment (typically 4 weeks). Consequently, the long-term, dose-dependent safety of esketamine, particularly regarding potential cognitive impairment or substance use disorders, could not be adequately assessed.

Fourth, there was heterogeneity in the reporting of certain AEs (e.g., somnolence) across trials. While we identified the administration route as a source of variance, differences in trial design, patient baseline characteristics, and clinical settings may still contribute to the observed heterogeneity.

Finally, the lack of individual-level patient data limited our ability to perform a detailed dose-response curve analysis, restricting our conclusions to comparisons between discrete dose groups (e.g., 56 mg vs. 84 mg).

## 6 Future perspectives

Our analysis underscores several avenues for subsequent investigation. To fully elucidate the long-term safety profile of esketamine, future research should prioritize long-term prospective trials with follow-up periods exceeding 1 year. Such studies should specifically focus on dose optimization strategies, such as intermittent administration to potentially reduce cumulative side-effect risks (Veraart et al., 2025). Moreover, the exploration of alternative administration routes, including subcutaneous injection, may offer a clinical pathway to minimize nasal spray–related adverse effects (Lucchese et al., 2021). Beyond administration methods, recent evidence highlights the potential safety benefits of esketamine metabolites, which may represent an innovative approach to achieving therapeutic efficacy while reducing treatment-emergent adverse events (TEAEs) (Horowitz and Moncrieff, 2021; Koutrouli et al., 2025). Integrating these strategies will be pivotal in refining the clinical utility of esketamine for TRD.

## 7 Conclusion

In conclusion, this meta-analysis demonstrates that esketamine is a rapid-acting treatment for treatment-resistant depression (TRD), improving remission rates compared with placebo. However, its safety profile exhibits a dose-dependent relationship. Our findings indicate that higher dosages (84 mg) are associated with an increased risk of transient adverse events, such as dissociation, nausea, and vertigo, leading to a 2.22-fold higher risk of treatment discontinuation DAE.

Our subgroup analysis indicates that both the efficacy and tolerability of esketamine are consistent across East Asian (Mainland China) and global/Western populations (P = 0.208), suggesting that global safety data can be extrapolated to East Asian clinical settings despite potential pharmacogenetic variations in *CYP2B6* activity. Our findings favors the intranasal route for its balanced risk-benefit profile.

Compared with previous reviews, this study provides granular evidence on the tolerability threshold of esketamine. Clinicians should adopt a personalized titration strategy, prioritizing the 56 mg dose to establish tolerability before escalating to 84 mg, while maintaining monitoring during the acute post-administration period to minimize the risk of treatment cessation.

## Data Availability

The original contributions presented in the study are included in the article/Supplementary Material, further inquiries can be directed to the corresponding author.
