# Supplementary material for: Dose-dependent adverse events of esketamine in treatment-resistant depression: a systematic review and meta-analysis of randomized controlled trials
Source: Front Pharmacol. 2026 May 28;17:1792570. doi: 10.3389/fphar.2026.1792570 (PMC13253624; doi:10.3389/fphar.2026.1792570)
Supplement: Supplementary file 1 [file Supplementaryfile1.docx]

search string：

The specific search strategy used for PubMed was as follows:((((((("toxicity" [Subheading]) OR ("adverse effects" [Subheading])) OR (((((((((((((("Risk Assessment"[Mesh]) OR (Risk Assessments[Title/Abstract])) OR (Assessment, Risk[Title/Abstract])) OR (Health Risk Assessment[Title/Abstract])) OR (Assessment, Health Risk[Title/Abstract])) OR (Health Risk Assessments[Title/Abstract])) OR (Risk Assessment, Health[Title/Abstract])) OR (Benefit-Risk Assessment[Title/Abstract])) OR (Assessment, Benefit-Risk[Title/Abstract])) OR (Benefit-Risk Assessments[Title/Abstract])) OR (Assessment, Risk-Benefit[Title/Abstract])) OR (Risks[Title/Abstract] AND Benefits[Title/Abstract])) OR (Risk Analysis[Title/Abstract])) OR (Risk Analyses[Title/Abstract]))) OR (((("Patient Safety"[Mesh]) OR (Patient Safeties[Title/Abstract])) OR (Safeties, Patient[Title/Abstract])) OR (Safety, Patient[Title/Abstract]))) OR (("Therapeutic Index, Drug"[Mesh]) OR (Drug Therapeutic Indices))) OR ((((((((((((((("Drug-Related TEAEs and Adverse Reactions"[Mesh]) OR (Drug Related TEAEs and Adverse Reactions)) OR (TEAEs of Drugs)) OR (Drug-Related TEAEs and Adverse Reaction)) OR (Adverse Drug Reaction)) OR (Adverse Drug Reactions)) OR (Drug Reactions, Adverse)) OR (Reactions, Adverse Drug)) OR (Adverse Drug Events)) OR (Drug Events, Adverse)) OR (Drug TEAEs)) OR (Effects, Drug Side)) OR (Drug Toxicity)) OR (Drug Toxicities)) OR (Toxicities, Drug))) AND ((((((("Treatment Adherence and Compliance"[Mesh]) OR (Therapeutic Adherence[Title/Abstract] AND Compliance[Title/Abstract])) OR (Treatment Adherence[Title/Abstract])) OR (Adherence, Treatment[Title/Abstract])) OR (Therapeutic Adherence[Title/Abstract])) OR (Adherence, Therapeutic[Title/Abstract])) OR (((("Drug Tolerance"[Mesh]) OR (Drug Tolerances[Title/Abstract])) OR (Tolerance, Drug[Title/Abstract])) OR (Tolerances, Drug[Title/Abstract])))) AND (((((((("Esketamine" [Supplementary Concept]) OR (L-Ketamine[Title/Abstract])) OR ((-)-Ketamine[Title/Abstract])) OR (S-Ketamine[Title/Abstract])) OR ((S)-2-(o-chlorophenyl)-2-(methylamino)cyclohexanone[Title/Abstract])) OR (Kataved[Title/Abstract])) OR (Spravato[Title/Abstract])) AND (((((((((((((((((((("Depressive Disorder, Treatment-Resistant"[Mesh]) OR (Depressive Disorders, Treatment-Resistant[Title/Abstract])) OR (Depressive Disorder, Treatment Resistant[Title/Abstract])) OR (Disorders, Treatment-Resistant Depressive[Title/Abstract])) OR (Disorder, Treatment-Resistant Depressive[Title/Abstract])) OR (Treatment-Resistant Depressive Disorder[Title/Abstract])) OR (Treatment-Resistant Depressive Disorders[Title/Abstract])) OR (Treatment Resistant Depression[Title/Abstract])) OR (Depressions, Treatment Resistant[Title/Abstract])) OR (Depression, Treatment Resistant[Title/Abstract])) OR (Resistant Depressions, Treatment[Title/Abstract])) OR (Resistant Depression, Treatment[Title/Abstract])) OR (Treatment Resistant Depressions[Title/Abstract])) OR (Refractory Depression[Title/Abstract])) OR (Depression, Refractory[Title/Abstract])) OR (Refractory Depressions[Title/Abstract])) OR (Therapy-Resistant Depression[Title/Abstract])) OR (Depressions, Therapy-Resistant[Title/Abstract])) OR (Therapy Resistant Depression[Title/Abstract])) OR (Therapy-Resistant Depressions[Title/Abstract]))).
