# Supplementary material for: Dose-dependent adverse events of esketamine in treatment-resistant depression: a systematic review and meta-analysis of randomized controlled trials
Source: Front Pharmacol. 2026 May 28;17:1792570. doi: 10.3389/fphar.2026.1792570 (PMC13253624; doi:10.3389/fphar.2026.1792570)
Supplement: Supplementary file 2 [file Table1.docx]

Supplementary Material Table 1 The main RCT list included in AJP 2024 (Fountoulakis et al.)

| Serial Number | Core Research (Author/Year) | Article Title | Eliminate the cause |
| --- | --- | --- | --- |
| 1 | Singh et al., 2016 | Intravenous Esketamine in Adult Treatment-Resistant Depression: A Double-Blind, Double-Randomization, Placebo-Controlled Study | The study was excluded based on our pre-specified exclusion criterion regarding **data integrity and extractability**. Specifically, Singh et al. utilized a **Sequential Parallel Comparison Design (SPCD)** and focused on **dosing frequency** (twice vs. thrice weekly) rather than discrete dose tiers. The complex double-randomization and the very small sample size (N=30) made it impossible to extract clean, non-confounded dose-specific AE incidence rates that are comparable with the other standard parallel-group RCTs in our analysis. |
| 2 | Canuso et al. (2018) | Efficacy and Safety of Intranasal Esketamine for the Rapid Reduction of Symptoms of Depression and Suicidality in Patients at Imminent Risk for Suicide: Results of a Double-Blind, Randomized, Placebo-Controlled Study | The study was excluded from our analysis for several methodological reasons. First, in terms of **Study Population**, this trial targeted patients with **MDD at imminent risk for suicide**, whereas our protocol focuses strictly on **Treatment-Resistant Depression (TRD)**. These populations exhibit distinct clinical characteristics and treatment histories. Second, regarding the **Dose-Dependent Analysis**, Daly 2018 utilized a single target dose of **84 mg** without parallel multiple-fixed-dose arms (e.g., 28 mg vs. 56 mg vs. 84 mg). Since our primary objective is to model the **dose-response relationship** for adverse events, this study did not provide the stratified data necessary for such a comparison. This decision aligns with our exclusion criteria regarding “population mismatch”and “inability to extract dose-specific safety data” |
| 3 | Popova et al., 2019 | Efficacy and Safety of Intranasal Esketamine Adjunctive to Oral Antidepressant Therapy in Treatment-Resistant Depression: A Randomized Phase 3 Study (TRANSFORM-2) | We appreciate the reviewer’s comment regarding Popova et al. (2019, TRANSFORM-2). While recognized as a pivotal trial, it was excluded from our meta-analysis based on our pre-specified criterion:Inability to extract dose-specific safety data.  Specifically, Popova et al. utilized a flexibly dosed regimen (56 mg or 84 mg), and the adverse events (AEs) were reported as a pooled total for the entire esketamine arm. Since our study’s primary objective is to conduct a granular dose-dependent analysis to identify “safety thresholds”, the lack of independent AE rates for the 56 mg vs. 84 mg subgroups precluded its inclusion. Furthermore, the simultaneous initiation of a new oral antidepressant in that study introduces confounding variables regarding the etiology of AEs, which would compromise the internal validity of our dose-response modeling. |
| 4 | Fedgchin et al., 2019 | Efficacy and Safety of Fixed-Dose Esketamine Nasal Spray Combined With a New Oral Antidepressant in Treatment-Resistant Depression: Results of a 4-Week, Randomized, Double-Blind Study (TRANSFORM-1) | Not ruled out（Reference 25 in the article） |
| 5 | Daly et al., 2019 | Efficacy of Esketamine Nasal Spray Plus Oral Antidepressant Treatment for Relapse Prevention in Patients With Treatment-Resistant Depression: A Randomized Clinical Trial (SUSTAIN-1) | Not ruled out（Reference 24 in the article） |
| 6 | Ochs-Ross et al., 2020 | Efficacy and Safety of Esketamine Nasal Spray Plus an Oral Antidepressant in Elderly Patients With Treatment-Resistant Depression (TRANSFORM-3) | Not ruled out（Reference 26 in the article） |
| 7 | Fu et al., 2020 | Intranasal Esketamine for Depressive Symptoms in Adults With Major Depressive Disorder and Active Suicidal Ideation With Intent (ASPIRE-II) | The study was excluded based on our protocol’s population and intervention criteria. First, the ASPIRE I trial enrolled patients with **Major Depressive Disorder (MDD) at acute suicidal risk**, which differs from the **Treatment-Resistant Depression (TRD)** population defined in our PICO framework. Second, our meta-analysis focuses on the **dose-dependent safety profile**, requiring studies with clear dose stratification. Since ASPIRE I utilized a single target dose of **84 mg** (with an SOC background) rather than a comparative multiple-fixed-dose design, it did not provide the granular data necessary for our dose-response modeling. This aligns with our exclusion criterion regarding the “inability to extract dose-specific safety data” |
| 8 | Ionescu et al., 2021 | Esketamine Nasal Spray for Rapid Reduction of Depressive Symptoms in Patients With Major Depressive Disorder Who Have Active Suicidal Ideation With Intent (ASPIRE-I) | 1.Population Heterogeneity: Our meta-analysis focuses specifically on Treatment-Resistant Depression (TRD). ASPIRE II recruited patients with MDD and acute suicidal ideation with intent (MDD-SI). The clinical urgency, hospitalization requirements, and prior treatment histories of MDD-SI patients differ significantly from the stable TRD population, which would introduce substantial clinical heterogeneity.  2. Absence of Multi-Dose Comparison: Our study aims to evaluate dose-dependent risks (e.g., 28 mg vs. 56 mg vs. 84 mg). ASPIRE II utilized a single target dose of 84 mg (plus Standard of Care) without a parallel dose-comparison design. Consequently, it does not provide the stratified data required for our dose-response modeling or subgroup analysis by dose tiers. |
| 9 | Takahashi et al., 2021 | Efficacy and Safety of Esketamine Nasal Spray Plus an Oral Antidepressant in Japanese Patients with Treatment-Resistant Depression: A Phase 2b/3 Study | Not ruled out（Reference 19 in the article） |
| 10 | Chen et al., 2022 | A Randomized, Double-Blind, Placebo-Controlled Study of Esketamine Nasal Spray Plus Oral Antidepressant in Chinese Patients with Treatment-Resistant Depression | Not ruled out（Reference 30 in the article） |
