# Supplementary material for: Dose-dependent adverse events of esketamine in treatment-resistant depression: a systematic review and meta-analysis of randomized controlled trials
Source: Front Pharmacol. 2026 May 28;17:1792570. doi: 10.3389/fphar.2026.1792570 (PMC13253624; doi:10.3389/fphar.2026.1792570)
Supplement: Supplementary file 3 [file Table2.docx]

Supplementary Material Table 2 Classification of discontinuation causes

| Outcome | No. of RCTs | RR (95% CI) | P-value | I^2^ |
| --- | --- | --- | --- | --- |
| All-cause Discontinuation | 9 | 1.15 (0.85, 1.56) | 0.36 | 0% |
| Discontinuation due to AEs | 9 | 2.22 (1.11，4.45) | 0.025 | 0% |
